# The relationship between self-report of depression and media usage

**DOI:** 10.3389/fnhum.2014.00712

**Published:** 2014-09-12

**Authors:** Martin Block, Daniel B. Stern, Kalyan Raman, Sang Lee, Jim Carey, Ashlee A. Humphreys, Frank Mulhern, Bobby Calder, Don Schultz, Charles N. Rudick, Anne J. Blood, Hans C. Breiter

**Affiliations:** ^1^Medill Integrated Marketing Communications, Northwestern UniversityEvanston, IL, USA; ^2^Applied Neuromarketing Consortium, Medill, Kellogg, and Feinberg Schools, Northwestern UniversityEvanston, IL, USA; ^3^Department of Psychiatry and Behavioral Science, Warren Wright Adolescent Center, Northwestern University Feinberg School of MedicineChicago, IL, USA; ^4^Laboratory of Neuroimaging and Genetics, Department of Psychiatry, Massachusetts General HospitalBoston, MA, USA; ^5^Department of Marketing, Kellogg School of Management, Northwestern UniversityEvanston, IL, USA; ^6^Department of Urology, Northwestern University Feinberg School of MedicineChicago, IL, USA; ^7^Mood and Motor Control Laboratory, Department of Psychiatry, Massachusetts General HospitalBoston, MA, USA

**Keywords:** depression, big data, marketing communications, media use

## Abstract

Depression is a debilitating condition that adversely affects many aspects of a person's life and general health. Earlier work has supported the idea that there may be a relationship between the use of certain media and depression. In this study, we tested if self-report of depression (SRD), which is not a clinically based diagnosis, was associated with increased internet, television, and social media usage by using data collected in the Media Behavior and Influence Study (MBIS) database (*N* = 19,776 subjects). We further assessed the relationship of demographic variables to this association. These analyses found that SRD rates were in the range of published rates of clinically diagnosed major depression. It found that those who tended to use more media also tended to be more depressed, and that segmentation of SRD subjects was weighted toward internet and television usage, which was not the case with non-SRD subjects, who were segmented along social media use. This study found that those who have suffered either economic or physical life setbacks are orders of magnitude more likely to be depressed, even without disproportionately high levels of media use. However, among those that have suffered major life setbacks, high media users—particularly television watchers—were even more likely to report experiencing depression, which suggests that these effects were not just due to individuals having more time for media consumption. These findings provide an example of how Big Data can be used for medical and mental health research, helping to elucidate issues not traditionally tested in the fields of psychiatry or experimental psychology.

## Introduction

Depression is known to affect many kinds of human behavior, and is quite common. As of 2005, the lifetime prevalence of major depressive disorder in the US population was reported to be 16.5% (Kessler et al., 2005a), with 6.7% prevalence in a 12-month period, 30.4% of which were severe (or 2.0% of the U.S. population) (Kessler et al., 2005b). Given the prevalence of depression, there is interest from a neuromarketing perspective in how it may be related to patterns of media consumption. Such issues are of fundamental concern for mechanisms of behavior change research and psychology (e.g., Morgenstern et al., 2013).

There is a developing literature evaluating the relationship between various types of media use and psychiatric conditions. For instance, one study found a high positive correlation between internet addiction and depression among university students (Orsal et al., 2012). Another study found that adults with major depressive disorder spent excessive amounts of leisure time on the computer, while those with dysthymia, panic disorder, and agoraphobia spent more time watching television than the control group or those with other disorders (de Wit et al., 2011). However, results have not always been consistent, particularly in the domain of social media use. A recent paper failed to find any association between social network use and depression in older adolescents (Jelenchick et al., 2013), while other studies have found positive associations between Facebook use and depression in high school students (Pantic et al., 2012), and Facebook use and a lack of subjective well-being in young adults (Kross et al., 2013). Given the heterogeneity across previous studies, and the rapid evolution of media formats over the past decade, we used a large consumer database (>19,000 subjects) to assess the relationship between self-reported depression (SRD) and media usage, taking into account demographic information which may impact the incidence of SRD such as employment status and disability. We used SRD since major depression cannot be diagnosed with big data surveys, and compared the rate of SRD to published incidence data on the diagnosis of major depression.

This study differed from previous studies in the following ways. (1) The sample size of the dataset was substantially larger than any previous study evaluating the relationship between media use and depression. (2) We evaluated the link between depression and multiple domains of media use, whereas most previous studies have focused primarily on single domains. For example, recent work with a smaller database has suggested there is an increase in digital media usage in “depressed” adolescents (Primack et al., 2009), but this study did not investigate its relationship to different subcomponents of media, such as social media, internet, and television.

Our analysis started with descriptive and bivariate statistical analyses. These were followed by omnibus approaches to assess general effects given the number of variables describing media usage: (a) Chi-squared Automatic Interaction Detection or CHAID tree analysis (Kass, 1980; Biggs et al., 1991) (a form of recursive partitioning; Zhang and Singer, 1999) and (b) discriminant analysis.

## Materials and methods

### Data acquisition

The dataset was derived from the Media Behavior and Influence Study (MBIS), a syndicated online study of American adult (i.e., ≥18 years of age) consumers, conducted twice yearly since 2002 by BIGinsight of Columbus, Ohio. The current wave of 19,776 participants was completed in December, 2012. Using a double opt-in methodology, each MBIS study was balanced to meet demographic criteria established by the US census. MBIS data has been used by a variety of well-known, commercial marketing organizations. Variables of interest included depression by gender, age, employment status, marital status, race and ethnicity, income, education, measures of isolation, and internet, TV and social media use. These variables were selected because they have been variables of interest in previous depression studies, and have been shown to have predictive value (e.g., Catalano and Dooley, 1977; Wilkowska-Chmielewska et al., 2013). Media usage for internet, television, and social media are based on yes/no responses to several day-parts of variable hour durations for a typical weekday (see Supplementary Materials). These blocks of time were shorter for typical waking hours and longer for overnight and weekend periods. Block length was used to weight media usage probability during the calculation of total hours of consumption (i.e., divided by the number of hours in each block of time). Average hour exposure probabilities were calculated for a 24 h period, and minutes per day were estimated by multiplying the result by 1440. Internet, TV, and social media usage were hence composite variables created as probabilities of number of minutes daily usage, derived from data indicating whether or not subjects used the respective services in seven discrete variable-hour blocks.

### Data analysis

Three types of analysis were performed with this data. First, we performed a descriptive statistical analysis, inclusive of correlations between depression and media consumption variables to facilitate interpretation of the subsequent analyses. Second, we used the results to inform a type of recursive partitioning (Morgan and Sonquist, 1963; Friedman, 1977; Breiman et al., 1984; Gordon and Olshen, 1984; Quinlan, 1986; Mingers, 1989), namely CHAID tree analysis (Kass, 1980; Biggs et al., 1991). Third, we performed a multivariate discriminate analysis. Given the descriptive statistical analyses were standard, these are not further discussed herein. In all analyses below excepting the CHAID analysis, fewer than 50 total comparisons were made; to correct for multiple comparisons we used a Bonferroni correction for 50 comparisons, requiring a *p* < 0.001 to be considered a significant result.

#### CHAID tree analysis

We performed two recursive partitioning analyses, one focused on SRD and the second on a variable not of interest, namely non-SRD, to act as a control for SRD results. Our working hypothesis was that the control analysis of non-SRD subjects would not replicate or provide an opponent (i.e., completely non-overlapping) set of nodes to the analysis of SRD subjects.

Construction of statistical CHAID trees (SPSS tree) evaluated the interaction among a number of predictor variables of SRD, and separately non-SRD. Typically, such schemes are defined in terms of demographic variables such as age and gender; however we have also included occupation, education, marital status and media use. Splitting criteria included minimum parent node size of 100 and child node size of 50, and a *p*-value threshold of 0.05. These splitting criteria were used for both CHAID analyses.

#### Discriminant analyses

Discriminant analysis was used to conduct a multivariate analysis of variance for the hypothesis that people who self-reported having depression would differ significantly from non-SRD subjects on a linear combination of eleven variables: income, internet usage, TV usage, social media usage, education, age, living in top 10 metropolitan area (MSA), gender, having children, employment status, and disability. The discriminant analysis was run using SPSS defaults, resulting in the canonical linear discriminant analysis. Depression was the binary dependent variable entered in the “group” dialog. The discriminating variables were entered together (i.e., not stepwise) in the variables subcommand. The discriminating variables income, internet usage, TV usage, social media usage, and education, all took on continuous values in the range from 0 to 1. “Living in top 10 MSA,” gender, employment status and disability were binary categorical variables while having children was ordinal. Overall, the data were complete with no missing values (i.e., every subject had every data point).

## Results

### Descriptive and correlation analyses

#### Geographic and temporal patterns

**Figure 1:**
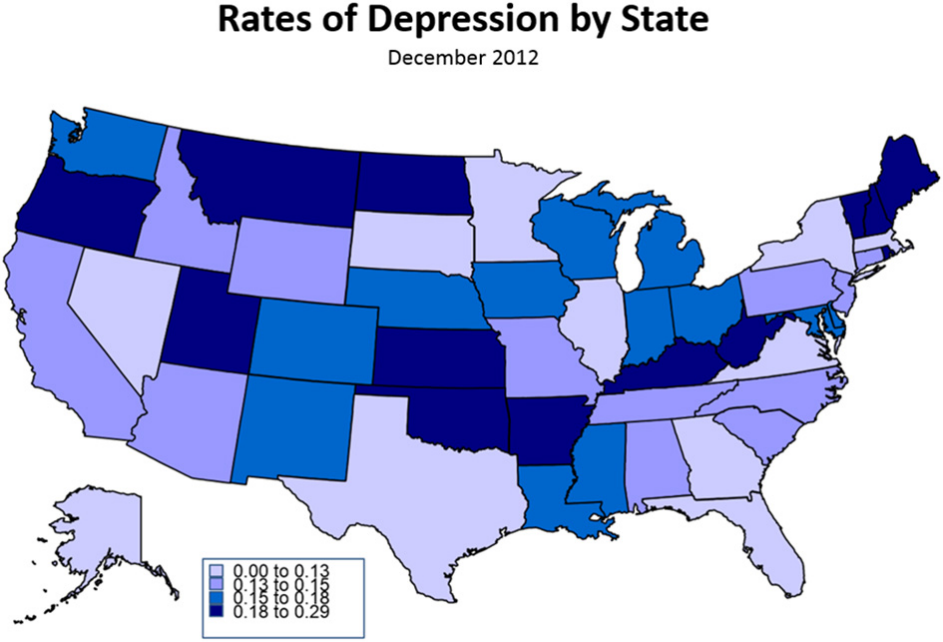
**Rates of Self-Reported Depression by State, December 2012**. This infographic characterizes rates of self-reported depression by state, with darker states showing greater rates of depression. The image demonstrates few patterns in depression by geography, with perhaps the exception that state with large metropolitan areas tend to show somewhat less depression.

The MBIS study shows little to no geographic pattern for SRD (Figure 1).

**Figure 2:**
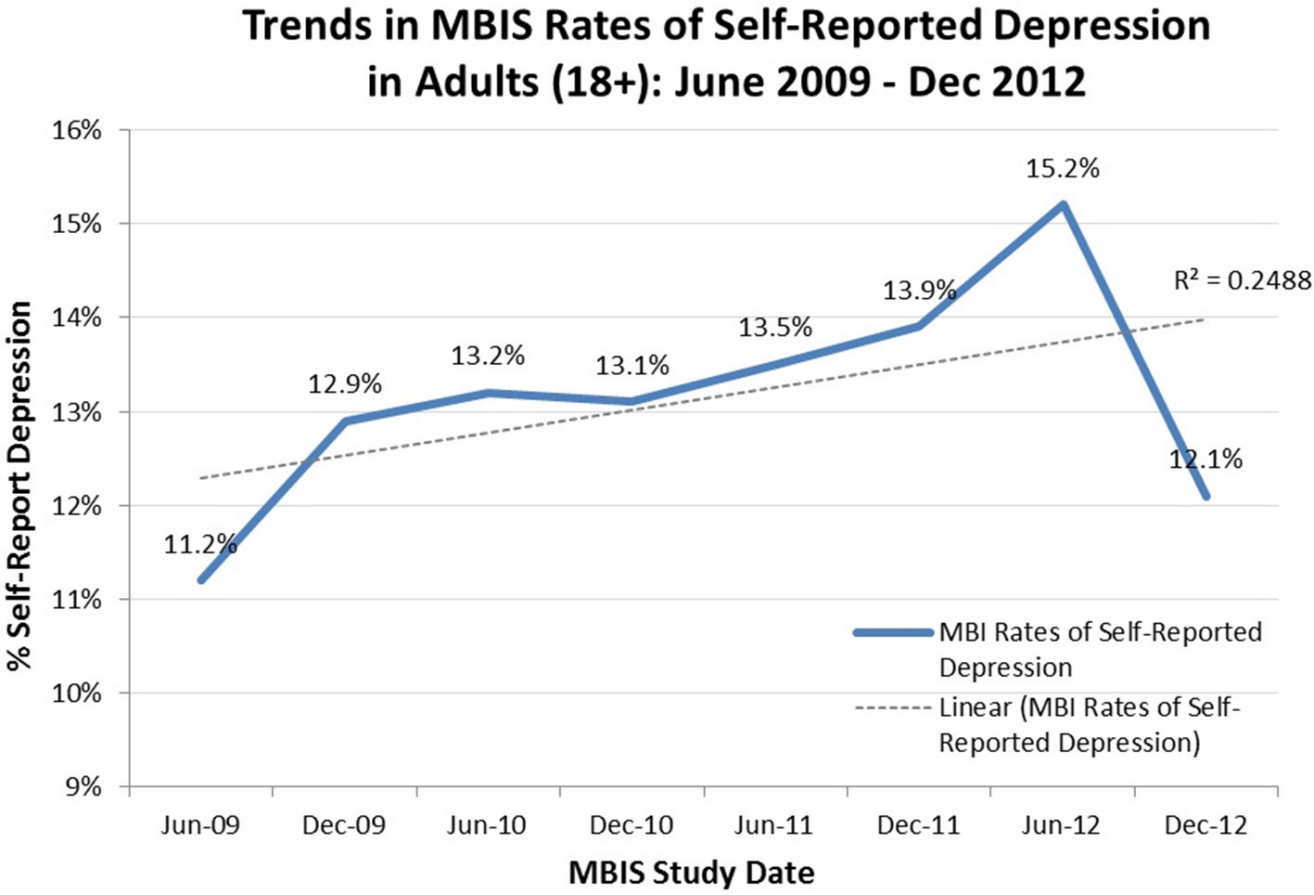
**Trends in MBIS Rates of Self-Reported Depression in Adults (18+), June 2009–December 2012**. This chart reports the rate of self-reported depression every 6 months, beginning in June 2009 and ending in December 2012, fitted with a linear trend line. All data was collected the same way by BIGinsight of Ohio as part of the MBIS study.

The data does show that SRD among all adults in the USA (18 and over) has grown from 11.2% in 2009 to 12.1% in December, 2012, with a linear trend (*r*^2^ = 0.246) (Figure 2). It is interesting that the rate rises to 15.2% in June 2012 (similar to rates in the 2005 MBIS data where the depressive rate was reported to be 14.9%), then drops to 12.1% in December 2012, which is consistent with a previous study that used the emotive content of tweets to show a similar annual pattern of decreased depression over Christmas (Dodds et al., 2011).

#### Depression demographics

***Gender***. Rates of SRD in the current study wave were nearly identical by gender as shown in Table 1, with males slightly lower at 11.8%, compared to females at 12.3%.

**Table 1:** **Depression by demographics, December 2012**.

	**% All adults**	**Adults with depression**	**Index**
Age (average years)^*^	45.4	43.8	96.3
Male	48.3	11.8	97.5
Female	51.7	12.3	101.7
Income (000)^*^	62.8	49.0	78.0
Have children	29.1	30.2	103.9
Live in top 10 MSA	24.7	10.0	82.6
Married	42.5	9.5	78.5
Living with unmarried partner	7.2	15.5	128.1
Divorced or separated	10.2	15.4	128.3
Widowed	3.0	12.4	102.5
Single, never married	25.7	14.1	116.5
Same sex union	0.5	22.2	183.5
Have not graduated high school	1.5	21.7	179.3
Graduated high school	16.8	13.1	108.3
Technical school or vocational training	5.7	13.8	114.0
1–3 years of college (did not graduate)	20.2	15.1	124.8
Associates or professional degree	8.9	13.2	109.1
Bachelor's degree	22.5	9.4	77.7
Post college study or degree	13.5	8.8	72.7
Business Owner	4.2	11.7	96.7
Professional/managerial	25.5	8.2	67.8
Salesperson	3.6	11.5	95.0
Factory worker/laborer/driver	3.3	9.6	79.3
Clerical or service worker	9.5	11.9	98.3
Homemaker	3.6	14.7	121.5
Student, high school or college	8.4	13.0	107.4
Military	0.7	11.6	95.9
Retired	13.7	10.8	89.3
Unemployed	5.5	18.8	155.4
Disabled (unable to work)	2.0	42.7	352.9
Obsessive-compulsive disorder (OCD)	2.1	9.8	458.1
Anxiety	12.9	54.8	425.8
Dyslexia	0.8	2.6	334.5
Fibromyalgia	2.3	7.4	327.5
Insomnia/difficulty sleeping	8.4	27.5	325.8
Restless leg syndrome(RLS)	4.1	11.6	279.8
Irritable Bowel Syndrome (IBS)/crohn's disease	2.4	6.3	262.6
Chronic bronchitis/COPD	2.9	7.3	252.5
Sleep apnea	6.6	16.4	248.2
Heartburn/indigestion	9.9	22.7	230.3
Headaches/migraines	14.0	29.6	211.5
Back pain	21.5	42.7	198.4
Acid reflux	15.8	30.5	192.9
Heart disease	3.2	5.9	185.9
Hearing impairment	4.3	8.0	184.6
Overweight	21.2	37.6	177.1
Arthritis	15.5	27.3	176.4
Asthma	9.7	17.0	174.8
Vision impairment	15.0	24.8	165.2
Enlarged prostate/Benign Prostatic Hyperplasia (BPH)	2.2	3.6	162.6
Diabetes	9.3	15.0	162.1
Osteoporosis	2.5	4.1	161.6
High cholesterol	18.8	29.3	155.9
Black	18.0	8.7	71.9
Asian	3.0	7.9	65.3
Multi	0.8	16.9	139.7
Native	0.4	15.5	128.1
White	58.4	13.6	112.4
Other	0.5	9.9	81.8
Hispanic	18.9	10.9	90.1

*The three columns of numeric values represent (i) the percentage of all adults in the survey with the given attribute (excepting income and age, which lists the survey mean), (ii) the percentage of subjects having the given attribute that self-reported depression, and (iii) the index of the given attribute as it relates to depression, which is calculated as the percentage of those depressed, divided by the total number depressed multiplied by 100. Rows on the left of the table are clustered around demographics, relationship status, education, and work identification. Rows on the right are clustered by illnesses separate from depression, and with race/ethnicity (Note: These do not follow NIH definitions of race and ethnicity)*.

***Age and marital status***. Bivariate analysis suggested an inverse linear association of SRD with age, which is consistent with previously reported studies (Henderson et al., 1998). Individuals who were married were also different than those who were unmarried as shown in Table 1, with married respondents representing a large portion of the sample (42.5%), and reporting a lower SRD rate of 9.5%. The highest rate of SRD was from those in same sex unions, at 22.2%. Those living with an unmarried partner, divorced or separated, or single (never married) reported rates between 14.1% and 15.5%, while those that were widowed reported rates (12.4%) nearly the same as the overall average.

***Race and ethnicity***. Table 1 showed lower rates among Hispanics (10.9%), and lower yet among African-Americans (8.7%) and Asians (7.9%) as compared to Multi-ethnic individuals and Caucasians. SRD was highest among Caucasians (13.6%), who represented more than half (58.4%) of the sample studied.

***Income and education***. Both income and education (Table 1), also demonstrated a strong inverse linear association with SRD, similar to age (statistics not provided given omnibus analyses to follow). Non-high school graduates self-reported a 21.7% depression rate compared to those with post college study or degree at 8.8%. The overall average income was $62,800, with those reporting depression indicating an average of $49,000. Occupation levels showed similar effects, as shown in Table 1, with those disabled (unable to work) reporting a 42.7% depression rate. Other high reporting categories included the unemployed at 18.8%, and students at 13.0%. The lowest category was professional and management at 8.2%.

***Health and lifestyle characteristics***. SRD was also related to the reporting of other health conditions as shown in Table 1. Generally, those reporting depression were likely to say they had other health-related conditions, such as anxiety (54.8%). Other conditions more prevalent in SRC subjects included: back pain (42.7%), overweight (37.6%), acid reflux (30.5%), headaches/migraines (29.6%), insomnia/difficulty sleeping (27.5%) (Table 1).

***Isolation***. Residents of states with large urban areas and those living in the top 10 metropolitan statistical areas (MSAs), have lower rates of SRD. The top 10 MSAs include Los Angeles, New York, Chicago, San Francisco, Philadelphia, Washington, Boston, Detroit, Phoenix and Houston. This suggests that residents of rural areas tend to report higher rates of depression.

***Media use***. Overall there were low but significant positive linear correlations between SRD and media consumption. In these descriptive analyses, the three most consumed media were television, on average 129 min per day per adult (18+), the internet, on average 143 min per day, and social media, on average 83 min per day. The bivariate association (*r*) between SRD and television consumption was 0.089, surfing the internet was 0.089 and social media was 0.063 (all *p* < 0.001).

**Figure 3:**
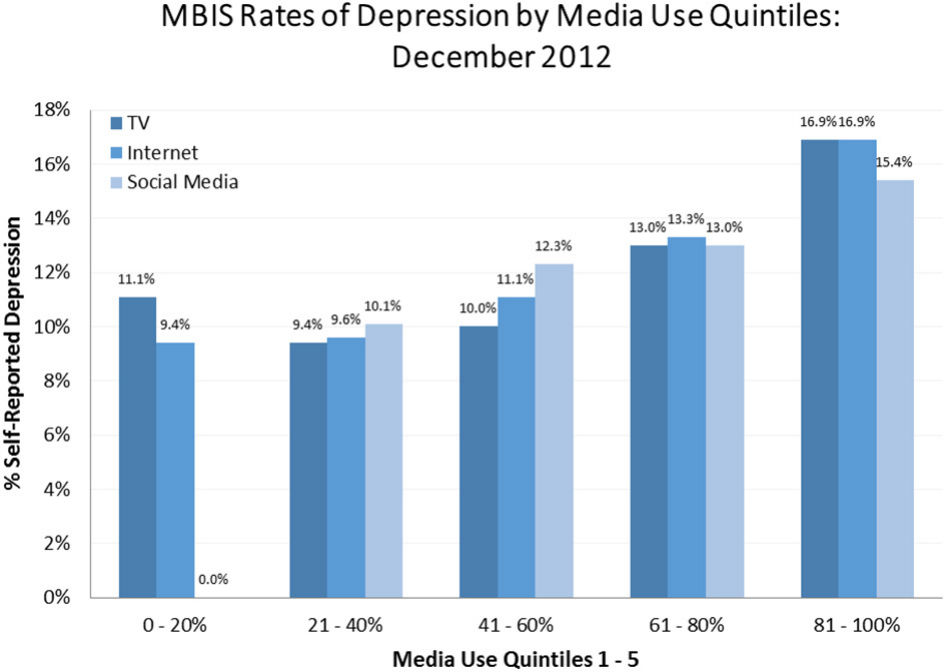
**MBIS Rates of Depression by Media Use Quintiles, December 2012**. This chart demonstrates the percent of subjects with depression in each media quintile. Quintiles were determined by ordering subjects based on estimated minutes of a given media consumed; the first 1/5 used the least of a given media and comprised the 0–20% quintile, the second fifth used more than the first 1/5 (but less than the third 1/5) and comprised the 21–40% quintile, and so on. Quintiles were computed for each type of media use of interest and graphed side by side. The graph depicts a clear trend associating increased media usage with increased rates of depression.

Media usage quintiles, a method commonly used in the media industry, were created using the composite media usage variables described above, and showed higher rates of depression among the most active users of media. Figure 3 shows that for the highest 20% of television users (quintile 5, 289 min per day) the SRD rate was 16.9%. The SRD rate among the highest internet users (327 min per day) was also 16.9%. SRD was slightly lower among the highest social media users (279 min per day) at 15.4%. The patterns among all three media categories were the same: higher consumption of any form of media was associated with higher rates of reported depression.

It should be noted that there was some co-linearity between the three media categories. The correlation of television and internet consumption was moderate at 0.495, slightly higher for internet and social media at 0.510, but lower for television and social media at 0.247. All of these correlations were significant (*p* < 0.001), raising the possibility of simultaneous consumption.

### CHAID tree analysis

**Figure 4:**
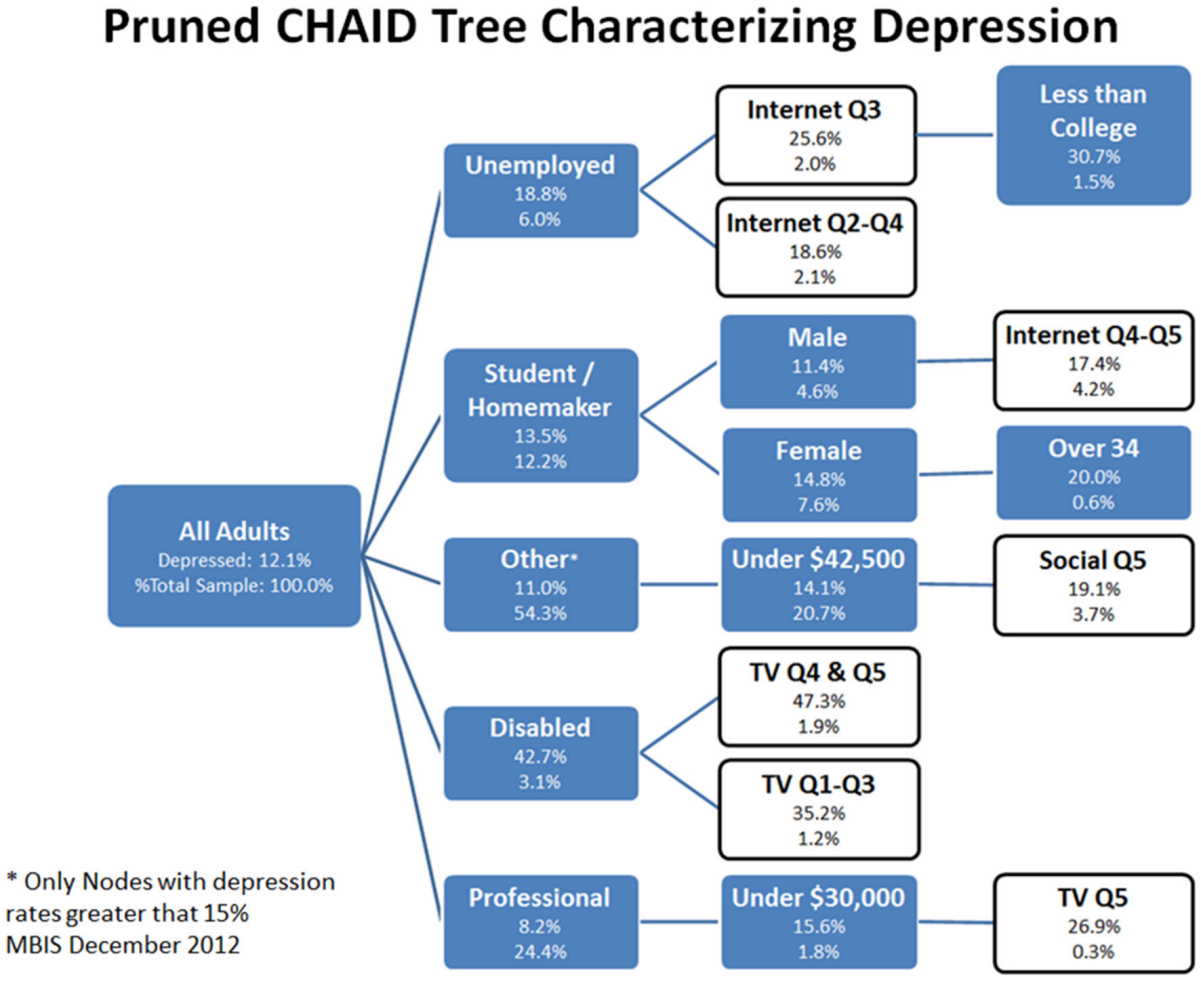
**Pruned CHAID Tree Characterizing SRD, December 2012**. The pruned CHAID tree shows groups of subjects wherein rates of depression were greater than 15%. These nodes represent only a subset of all nodes generated by the CHAID tree. Of particular interest for this paper are nodes that are white (instead of blue); these nodes have been highlighted because they are partially defined by the presence of a media use quintile.

**Figure 5:**
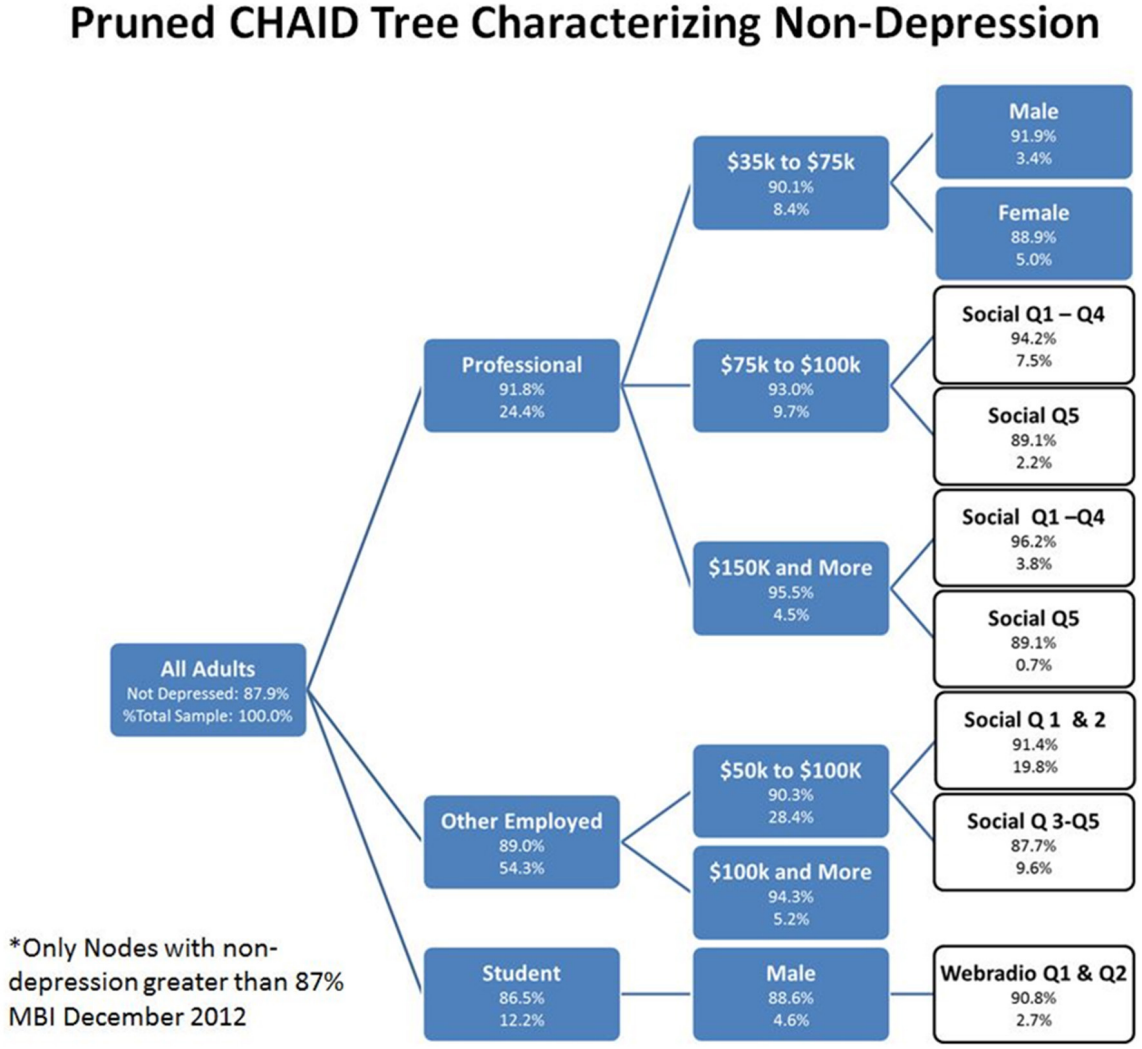
**Pruned CHAID Tree Characterizing Non-SRD, December 2012**. The pruned CHAID tree shows groups of subjects wherein rates of non-depression were greater than 87%. These nodes represent only a subset of all nodes generated by the CHAID tree. Of particular interest for this paper are nodes that are white (instead of blue); these nodes have been highlighted because they are partially defined by the presence of a media use quintile.

The analyses reported above were limited to bivariate correlations. To better understand how multiple variables for media consumption and other demographics/activities related to SRD, a multivariate segmentation scheme was employed based on recursive partitioning (Morgan and Sonquist, 1963; Friedman, 1977; Breiman et al., 1984; Gordon and Olshen, 1984; Quinlan, 1986; Mingers, 1989). The first CHAID tree (Kass, 1980; Biggs et al., 1991) (Figure 4) shows the interaction among the predictor variables on the rate of SRD (the target variable). The second CHAID tree (Figure 5) shows the interaction among the predictor variables and those who did not self-report being depressed. The first analysis on depressed individuals generated 22 terminal nodes, while the second on non-depressed subjects generated 21. The trees (Figures 4, 5) were pruned to include only 8 and 10 terminal nodes where the depression rate was 15% or more and the non-depression rate was 87% or higher, respectively. The tree nodes showed the variable used to create the node, the depression rate, and the percent of all adults that the node represented. In Figure 4, those that were unemployed, for example, were 6.0% of the sample and reported a depression rate of 18.8%. Note that media-related nodes were shown in white and other variables shown in blue/gray.

In the analysis of SRD subjects, the CHAID tree segments (Figure 4) that were the basis for understanding the relationship of depression to media and other variables were as follows. In general, factors such as disability, unemployment and lower incomes were associated with higher rates of SRD. Media consumption tended to significantly leverage the rate attributable to these characteristics. Six nodes of interest are briefly described. The node with the highest depression rate (47.3%) was being disabled (1) and in the top two TV consumption quintiles. This was compared to being disabled and in the bottom three TV quintiles with a somewhat lower depression rate (35.2%). The next highest depression node (30.7%) consisted of (2) those who were unemployed, in the top internet quintile, and had less than a college education. Those in a professional or managerial occupation that made $30,000 or less, and were in the highest TV quintile (3), reported a depression rate of 26.9%. (4) Female students or homemakers older than 34, reported a 20.0% depression rate. (5) Those in other occupations, including workers, sales, military and retired, that make less than $42,500 and were in the highest social media quintile, reported a depression rate of 19.1%. (6) Male students or homemakers in the highest two internet quintiles reported a depression rate of 17.4%.

In the analysis of non-depressed individuals (non-SRD), the CHAID tree segments (Figure 5) that best explained the relationships between media use, demographic variables, and non-SRD, described ten nodes. The node with the highest non-depression rate (96.2%) was being professional (1) with a salary of $150,000 and more in the lowest social media quintiles. This was compared to being professional (2) with a salary of $150,000 and more in the highest social media quintile (89.1%). The next highest non-depression node (3) consisted of those in other occupations, including workers, sales, military and retired, making $100,000 and more (94.3%). This was contrasted with (4) those in other occupations making $50,000 to $100,000 and in the lowest social media quintiles (91.4%), and (5) those in other occupations making $50,000 to $100,000 and in the highest social media quintiles (87.7%). Those who were professional making $75,000 to $100,000 and in the lowest social media quintile had a non-depression rate of 94.2% (6), whereas those who were professional making $75,000 to $100,000 and in the highest social media quintile had a non-depression rate of 89.1% (7). Professionals making $35,000 to $75,000 and male gender (8) were higher (91.9%) than those professionals making $35,000 to $75,000 and female gender (9). Lastly, students who were male in the lowest web-radio quintile (10) had a non-depression rate of 90.8%. In general, being a student or employed with a high income were most closely associated with not being depressed, particularly when combined with varying levels of social media use.

It is important to note that the CHAID analysis with non-SRD did not replicate the analysis with SRD. Furthermore, there was a segmentation observed between these analyses which was distinct, in that the types of media use that segmented the SRD subjects was not the same as that which segmented the non-SRD subjects. The terminal nodes of the two analyses were different along dimensions of occupation, income, and media use.

### Discriminant analysis

The results of the discriminant analysis revealed that, other than disability and income, the three single best predictors of depression in this model were increased use of television, the internet, and social media (Table 2). The overall Chi-squared test of the discriminant model was significant (Wilk's λ = 0.945, Chi-square = 922.117, df = 9, Canonical correlation = 0.235, *p* < 0. 001). The structure matrix demonstrated the weights of the discriminating variables, an indication of their importance for predicting depression: being disabled (0.760), income (−0.519), internet consumption (0.399), television consumption (0.368), social media use (0.278), level of education (−0.255), being unemployed (0.223), age (−0.170), top 10 MSA (−0.142), female gender (0.062), and having children (0.010).

**Table 2:** **Structure matrix of discriminant analysis predicting depression, December 2012**.

**Canonical correlation 0.235 Wilk's lambda 0.945**	**Chi-square 922.117 significance <0.001**
Disabled	0.760
Income	−0.519
Internet usage	0.399
TV usage	0.368
Social media usage	0.278
Education	−0.255
Unemployed	0.223
Age	−0.170
Living in top 10 MSA	−0.142
Female	0.062
Having children	0.010

*This table reports the structure matrix of the discriminant analysis. The nine predictive variables used in the discriminant analysis are reported in the left column, while a measure of that variable's predictive importance in the discriminant function is listed on the right. A variable's importance is determined by its magnitude, while its relationship to depression is determined by its valence. Negative numbers describe an inverse relationship: for example, higher income is predictive of less depression*.

## Discussion

The primary finding of this study is that those who tend to use more media in general, also tend to have more self-reports of depression. We found a current incidence of SRD at 12.1% which is slightly less than reports of lifetime clinical depression and more than the 12 month incidence of diagnoses of major depression. However, the picture is far more nuanced than simple description of descriptive statistics and bivariate correlations between media use and depression. For instance, the CHAID tree analysis with SRD subjects (along with the discriminant analysis) shows that those who have suffered either economic or physical life setbacks are orders of magnitude more likely to be depressed, even without disproportionately high levels of media use (37.2%). However, among those that have suffered major life setbacks, high media users—particularly television watchers—were even more likely to report experiencing depression (47.3% in the highest two quintiles, as compared with 35.2% in the lower three quintiles), which suggests that these effects were not just due to individuals having more time for media consumption. These effects were not observed with the control analysis in non-SRD subjects. That the economically disadvantaged are significantly more likely to experience depression is well supported by research in social psychology, which suggests that lower-income groups feel a sense of disempowerment (Henry, 2005; Stephens et al., 2007). The lack of financial and temporal resources they experience can lead to feelings of a lack of control over one's life and an inability to act efficaciously in the world, which is thought to be a basic human need. Supporting this interpretation, the CHAID analysis of the non-SRD subjects showed that high earners that use less social media tend to be significantly less depressed.

Life challenges may not be the only experiences related to depression. As noted with our descriptive statistical analysis, persistent environmental factors such as isolation can also contribute to the prevalence of a psychological experience. Generally, isolation is a known correlate of depression symptomology, and our data suggest that residents of rural areas tend to report higher rates of depression. Within the context of isolation, one can distinguish between physical and non-physical isolation; and within non-physical isolation one can look at social and emotional isolation. These various subclasses of isolation find ample support in the literature. Weiss (1973) first distinguished the two types of non-physical isolation—social and emotional—which have subsequently been empirically shown as distinct (DiTommaso and Spinner, 1997). Although conceptually distinct, the various types of isolation interact. Physical isolation has been shown to affect social and emotional isolation, especially for the elderly (Dugan and Kivett, 1994) and adolescents (Brage et al., 1993). We measured social isolation through the proxy of living alone and physical isolation through the proxy of place of residence, finding that both correlate to rates of self-reported depression. For instance, those living in more populated cities (top 10 MSAs) tend to report lower rates of depression.

In addition to the current state of depression, the data we analyzed reveals that SRD has been in a state of flux over the past decade. At the beginning of this time frame, the rates we observed were low compared to 2005 MBIS data where the depressive rate was reported to be 14.9%; a figure consistent with a co-occurring 2005 study wherein a lifetime prevalence rate of 16.5% for major depressive disorder was reported (Kessler et al., 2005a,b). Interestingly, the 2005 MBIS data and the Kessler et al. (2005a,b) data show remarkable concordance despite differences in inclusion criteria (exclusion of non-English speakers in the Kessler et al., 2005a,b studies), the use of a structured clinical interview vs. self-report data, and overall subject demographics. This flux in reported incidence of depression over the past decade is further supported by the MBIS data showing self-reported depression has been on the rise in adults (18+) over the last 4 years in the United States.

It is worth considering the demographics of individuals (e.g., gender) reporting SRD in the context of a flux in depression rates over time. As recently as 5 years ago, females were more likely to report being depressed (i.e., SRD). However, in the most recent MBIS study, the data shows SRD to be similarly associated with both genders, with males reporting only a slightly lower rate of depression. This is different than the rates reported by Primack et al. (2009) where females were shown to be significantly more likely to be depressed, as was also observed in the December 2005 MBIS data. In comparison to prior big data reports, there appears to be a narrowing in the gap of reported depression in females and males, which could potentially reflect a change in the likelihood of genders to self-report. One factor that has remained constant was that depression is inversely related to age, with those younger than 24 reporting the highest rate, and older married persons reporting the lowest.

There are several important limitations to this study that are worth mentioning. First, the data used was self-reported depression, which does not necessarily reflect whether the subject has ever received a clinical diagnosis of depression. The subjective phenotypes of those who have a clinical diagnosis of major depression versus those that self-report depression could skew the data in a number of different ways. For instance, it has been observed that those who have been diagnosed with depression are sometimes reticent to share their diagnosis. Alternatively, there is a multiplicity of reasons to think that subjects without depression may report being depressed. The balance of these considerations leaves uncertainty in the true sample parameters, although the percentage of subjects with SRD in this study was quite similar to rates of depression found in previous studies.

Second, the variables computed for amount of television, internet, and social media use are not direct measures. These variables are composite variables computed from self-reports of whether or not subjects used those various media during discrete variable-hour-length blocks. This can introduce inter-subject variability along a number of dimensions. For instance, some subjects may report “yes” for one of the intervals based on an hour's worth of use, while others may respond the same based on several hours' worth of use. The probabilities computed represent just that, a probability of time spent using a given media relative to other subjects.

Third, the analyses done cannot speak to a causal relationship between media consumption and depression, or to any directionality between the observed associations. We think the likeliest explanation is that these two variables form a complex bi-directional relationship with autocatalytic properties. An alternative explanation is that depression and increased media use are a byproduct of a third confounding factor. It should also be noted that the direction of causality between depression and media use could also vary across individuals (i.e., whether media usage helps to ameliorate depression or whether it contributes to it). Whatever the exact relationship between depression and increased media use, it is clear that the two are closely associated.

Fourth, it is important to acknowledge the potential confounds of concurrent medical illness on assessing associations with SRD. In the literature on major depression, hypotheses have been raised that depression in association with a medical illness does not necessarily reflect the same structural and functional circuitry alterations seen in depression with strong familial heritability (e.g., see Cloninger, 2002; Breiter and Gasic, 2004; Breiter et al., 2006). There is a strong possibility of biological subtypes in depression (e.g., see Blood et al., 2010), meaning depression comorbid with other illnesses may reflect a directionality with media that is distinct from other putative depressive subtypes. Depression in association with another medical (e.g., severe coronary artery disease) or psychiatric condition (e.g., OCD, generalized anxiety, or body image disorders) may have a complex directional relationship with these other conditions, and there is published evidence that TV viewing itself is associated with anxiety and body image issues (e.g., see Thompson and Heinberg, 2002; de Wit et al., 2011), potentially leading to the self-reported depression. These issues also relate to the potential for drug and alcohol to confound effects with SRD; this data set did not contain such information, so future work is needed to assess the relationship of drug and alcohol effects on SRD and media use.

This information can help to form hypotheses to test in future studies of relevance to psychology. One such hypothesis could relate to the directionality of the relationship between SRD and media, to determine if any media use acts as feedback to exacerbate symptoms. Another hypothesis might attempt to relate the relationship to existing social psychological constructs such as the “empty self” hypothesis. Cushman (1990) developed the “empty self” hypothesis to describe those who feel depressed and may be likely to engage in impulsive or excessive consumption behavior in order to “fill up” a perceived deficiency in the self (see also Ahuvia, 2005). In the context of media usage, the “empty self” might be expected to show increased consumption of media associated with increased SRD; such behavior might be indicative of a subtype of major depression. A third possibility, is a hypothesis that increased media use by SRD subjects acts as indicator of the illness, much like a biomarker. Such hypotheses point to further opportunities for use of big data with psychological research.

In summary, the data reveal that there is a consistent pattern of results that link self-reported depression with increased media use, even when taking into account other variables, such as disability and unemployment. This media use was focused more on internet use and TV exposure, for those making self-reports of depression. The rate of SRD was between two standard indices used in published reports of clinically diagnosed major depression, namely the lifetime prevalence, and recent 12 month incidence of major depression. These observations suggest the current findings with big data may have relevance to the literature focused on the clinical diagnosis of depression.

### Conflict of interest statement

The authors declare that the research was conducted in the absence of any commercial or financial relationships that could be construed as a potential conflict of interest.
