# Supplementary material for: The relationship between self-report of depression and media usage
Source: Front Hum Neurosci. 2014 Sep 12;8:712. doi: 10.3389/fnhum.2014.00712 (PMC4162355; doi:10.3389/fnhum.2014.00712)
Supplement: Supplementary file 1 [file DataSheet1.DOCX]

**Supplementary Materials**

1. **Methodology for MBIS study from Prosper**

Q: What is the Margin of Error?

For the Monthly Consumer Survey and Media Behaviors & Influence™ Study, the margin of error averages +/- 1%. The margin of error is based on the large sample sizes Prosper is able to collect.

Q: How does Prosper use technology to improve reliability?

Prosper employs computer intensive statistical methods that our founders pioneered in the late 1980’s. These techniques not only balance our large sample of the survey results to the U.S. population, but our proprietary software also dynamically rebalances each cross-tab of the data in the Monthly Consumer Survey & Media Behaviors & Influence™ Study data. A technical review of these methods was conducted by Dr. Jerry Friedman of Stanford University, former Chairman of the Statistics Department.

Q: How is the data in the Monthly Consumer Survey & Media Behavior & Influence™ Study representative of the U.S. population?

In order to ensure accurate representation of all consumer groups, Prosper syndicated data is balanced to the U.S. Census simultaneously by 14 age/sex cells—7 age groups for females and 7 age groups for males. For each cross-tab, the data is dynamically rebalanced by how each subset differs from the overall sample and the U.S. population.

Q: What real world examples are there of Prosper data reliability, and does the Prosper data reflect known market realities?

Yes. We regularly conduct analysis on our syndicated data and track it against what others are finding as well as what is going on in the market. Furthermore, the National Retail Federation (NRF) relies on our data when they issue their retail industry press releases covering sales forecasts for various holidays throughout the year. Not only does the NRF and their members rely upon the information but so too does the Wall Street community in making large investment decisions. In addition, we work with the largest retailers and CPG companies in the world and they are continuously validating our data to make strategic decisions impacting significant dollar volumes.

Q: Why does Prosper collect surveys via email, as opposed to other methods?

Email based research, combined with the Prosper balancing mathematics, is currently the most accurate and cost-effective method of doing research. In 2013, the Pew Trust estimated that 83% of the population uses email or the internet at least occasionally. The Central Intelligence Agency last estimated in 2009 that there were 245 million internet users in the United States which equals a 77% penetration of today’s population. The Central Intelligence Agency also estimates that there are 139 million main telephone lines in the U.S. In addition, approximately 27% of U.S. households are now cell phone only with no land lines, making respondents much more difficult to reach by telephone. Concerning low income consumers, email is the last “free service”. Yahoo!, Gmail, and Hotmail all offer free email accounts. Free Internet access is available at most libraries and many community centers.

Q: Are there quality controls for panelist responses?

To ensure data quality, Prosper has a variety of controls in place. First and foremost, the company has advocated short and simple surveys since it was founded in 2000. Complicated and complex surveys also cause fatigue. Prosper recommends that all clients stay away from “mind-stopping” questions that cause respondents to have to stop and think about obscure details. In addition to short simple surveys, Prosper advocates not requiring answers unless they are needed for branch & skip logic. To ensure that surveys are completed with the necessary number of completes, Prosper over-collects each survey and runs a computer program analyzing the number of possible data points in the survey against the number collected in each respondent record. Those records that are deemed to be incomplete are thrown out and not counted in the final data.

Prosper’s experience with syndicated studies, best practices have been developed that can also be applied to custom surveys and proprietary client panels. The efficient and accurate methods used by Prosper allow clients to conduct custom surveys in a timely and cost-effective manner. As with our syndicated studies, the Prosper advantage makes gathering from our communities fast, inexpensive, and robust.

Q: Can Prosper conduct a custom survey from beginning to end?

Yes. Prosper provides top-to-bottom service from questionnaire development, survey programming and hosting, to analyzing the data results. The same InsightCenter software used with our syndicated studies is also available for use with custom survey analysis. We also have a network of Subject Matter Experts to bring in on special projects, such as those requiring discrete choice analysis or predictive modeling, which helps to keep costs down.

Q: Do you balance custom surveys to the U.S. population as you do with your syndicated studies?

Sometimes. The data can be balanced to the U.S. population only when the survey objective allows us to survey all Adults 18+ and does not include any screeners which disqualify respondents from participating. Survey data collected from groups with unknown incident rates cannot be balanced to the U.S. population. Though custom survey databases are often not balanced to the U.S. Census, the data is still dynamically balanced to the overall respondent group by age and gender. The dynamic balancing refines the data when cross-tabbing in order to reflect differences in age and gender among sub-groups of the overall sample. While balancing to the U.S. Census shifts age and gender proportions to reflect the population, balancing to the raw respondent group in a custom survey database is simply a fine-tuning of the data.

Q: What type of experience does Prosper have doing custom surveys?

Prosper has experience in many different research methodologies including, but not limited to: discrete choice modeling; price elasticity models; radio, television and print ad testing; advertising awareness & recall; package testing; monadic, proto-monadic and sequential monadic concept testing; qual-quant research; brand equity/health tracking; customer satisfaction; Net Promoter® analysis.

1. **Questions asked on the survey**

What year were you born?

Do you have any children?

Zip Code:

What is your gender?

1. male
2. female

What is the highest level of formal education you have completed?

1. Have not graduated high school
2. Graduated high school
3. Technical School or Vocational Training
4. 1 – 3 years of college (Did not graduate)
5. Associates or Professional Degree
6. Bachelor’s Degree
7. Post College Study or degree

Which one of the following categories best describes your current occupation?

1. Business Owner
2. Professional/Managerial
3. Salesperson
4. Factory Worker / Laborer / Driver
5. Clerical or Service Worker
6. Homemaker
7. Student, High School or College
8. Military
9. Retired
10. Unemployed
11. Disabled (Unable to work)
12. Other (please specify)

What is the annual total income of your household?

1. Less than $15,000
2. $15,000 to $24,999
3. $25,000 to $34,999
4. $35,000 to $49,999
5. $50,000 to $74,999
6. $75,000 to $99,999
7. $100,000 to $149,999
8. $150,000 or more

On an average WEEKDAY (MONDAY – FRIDAY), during which hours do you… (Check all that apply. If you don’t use a particular media in a daypart please leave that box blank)

1. Watch TV
   1. 6am – 10am
   2. 10am – Noon
   3. Noon – 4:30pm
   4. 4:30pm – 7:30pm
   5. 7:30pm – 11pm
   6. 11pm – 1am
   7. 1am – 6am
2. Surf the Internet
   1. 6am – 10am
   2. 10am – Noon
   3. Noon – 4:30pm
   4. 4:30pm – 7:30pm
   5. 7:30pm – 11pm
   6. 11pm – 1am
   7. 1am – 6am
3. Social Media/Instant Message
   1. 6am – 10am
   2. 10am – Noon
   3. Noon – 4:30pm
   4. 4:30pm – 7:30pm
   5. 7:30pm – 11pm
   6. 11pm – 1am
   7. 1am – 6am

On an average WEEKEND DAY (SATURDAY – SUNDAY), during which hours do you… (Check all that apply. If you don’t use a particular media in a daypart please leave that box blank)

1. Social Media/Instant Message
   1. 6am – 10am
   2. 10am – 3pm
   3. 3pm – 7pm
   4. 7pm – Midnight
   5. Midnight – 6am
2. Surf the Internet
   1. 6am – 10am
   2. 10am – 3pm
   3. 3pm – 7pm
   4. 7pm – Midnight
   5. Midnight – 6am
3. Watch TV
   1. 6am – 10am
   2. 10am – 3pm
   3. 3pm – 7pm
   4. 7pm – Midnight
   5. Midnight – 6am

Which of the following health related conditions do you suffer from? (Check all that apply):

1. Acid Reflux
2. Allergies
3. Anxiety
4. Arthritis
5. Asthma
6. Back Pain
7. Chronic Bronchitis/COPD
8. Depression
9. Diabetes
10. Dyslexia
11. Enlarged Prostate/BPH
12. Fibromyalgia
13. Hearing Impairment
14. Headaches/Migraines
15. Heartburn/Indigestion
16. Heart Disease
17. High Blood Pressure
18. High Cholesterol
19. Insomnia/Difficulty Sleeping
20. IBS/Crohn’s
21. Obsessive-compulsive disorder (OCD)
22. Osteoporosis
23. Overweight
24. Restless Leg Syndrome (RLS)
25. Sleep Apnea
26. Vision Impairment
27. None of the above
